# Beyond the jaw: vocal, oropharyngeal and cognitive-linguistic biomarkers for precision pain phenotyping in TMD

**DOI:** 10.22514/jofph.2026.056

**Published:** 2026-09-12

**Authors:** Candice J Adams-Mitchell, Jacob Koshy, Rory Reever, Amir Shaikh, Patricia Lopes Cabrera, Selenia Rubio, Margarete C. Ribeiro-Dasilva, Roger B. Fillingim

**Affiliations:** ^1^Department of Speech, Language and Hearing Sciences, College of Public Health and Health Professions, University of Florida, Gainesville, FL 32610, USA; ^2^Pain Research and Intervention Center (PRICE), University of Florida, Gainesville, FL 32611, USA; ^3^Department of Community Dentistry and Behavioral Science, College of Dentistry, University of Florida, Gainesville, FL 32610, USA; ^4^Division of Prosthodontics, Restorative Dental Sciences Department, College of Dentistry, University of Florida, Gainesville, FL 32611, USA

**Keywords:** Temporomandibular disorders, Vocal biomarkers, Speech acoustics, Swallowing, Cognitive function, Pain phenotyping

## Abstract

Temporomandibular disorders (TMDs) represent a group of conditions affecting the temporomandibular joint and its associated musculature. TMD is the leading cause of chronic orofacial pain. Current assessment relies heavily on patient-reported outcomes, which capture pain intensity and its impact but provide limited mechanistic or functional insight. This narrative conceptual review proposes a framework for investigating vocal, oropharyngeal, and cognitive-linguistic (VOCL) features as candidate behavioral biomarkers for precision pain phenotyping in TMD. We synthesize the literature from TMD and related neurological and chronic pain conditions to examine how alterations in neuromuscular control, autonomic regulation, and cognitive burden could plausibly manifest as changes in acoustic voice metrics, motor speech performance, swallowing function, and cognitive-linguistic tasks. Given the limited TMD-specific empirical evidence available to date, we outline a staged validation roadmap that specifies TMD subgroups, study designs, confounder control strategies, and reliability considerations necessary before clinical application. While promising, VOCL features should be regarded as investigational constructs requiring rigorous validation. Establishing standardized protocols and integrating VOCL measures with psychological and physiological domains may clarify the potential role of VOCL in TMD phenotyping and monitoring.

## 1. Introduction

Temporomandibular disorders (TMDs) are a group of more than 30 conditions that can cause pain and dysfunction in the jaw joint and the muscles responsible for jaw movement. TMD is the leading cause of chronic orofacial pain and, second only to back pain, is among the most common and debilitating musculoskeletal pain conditions [1]. In the United States, clinically diagnosed TMD affects approximately 5% to 12% of the population [2, 3, 4]. The actual prevalence of TMD is likely higher due to the underreporting of individuals who live with these disorders and choose not to seek treatment. The peak prevalence of TMD occurs during early-to-mid adulthood, and women are more frequently affected than men [1]. The estimated annual cost of TMD in the United States is approximately $4 billion, and TMD can result in serious health consequences [5]. Diagnosis and management of TMD remain challenging, with existing treatments showing limited efficacy and potentially significant adverse effects. One factor contributing to suboptimal TMD management is the absence of noninvasive biomarkers that could enhance the assessment of TMD.

In this article, after briefly defining TMDs and reviewing their health consequences, we discuss the potential value of exploring vocal, oropharyngeal, and cognitive-linguistic (VOCL) biomarkers for TMD assessment and management. We synthesize current evidence on how vocal, linguistic, motor speech, and swallowing features could serve as non-invasive indicators of pain phenotypes and treatment outcomes. By integrating evidence from speech-language pathology, pain science, and bioinformatics, we highlight the potential for VOCL biomarkers to improve diagnostic accuracy and support personalized TMD management.

This manuscript presents a narrative conceptual review that examines existing literature and proposes a framework for investigating vocal, oropharyngeal, and cognitive-linguistic (VOCL) features as potential behavioral biomarkers in temporomandibular disorders (TMD). Relevant literature was identified through targeted searches of PubMed, Scopus, and Google Scholar using combinations of terms including “temporomandibular disorders”, “voice biomarkers”, “speech acoustics”, “diadochokinetic (DDK) rate”, “dysphagia”, “cognitive function”, “chronic pain”, and “digital biomarkers”. Because this topic spans several disciplines and remains an emerging area of research, both foundational and recent studies were considered. Evidence from TMD as well as related neurological, psychiatric, and chronic pain conditions was included to help inform potential mechanisms linking VOCL measures with pain expression. This review was not conducted as a systematic or scoping review; rather, it aims to present a conceptual framework that may help guide future hypothesis-driven research.

## 2. TMD as a complex pain condition

TMDs are now understood to be far more complex than localized pain conditions affecting the masticatory system. The development and severity of these conditions are influenced by biological, psychological, and social factors [6, 7]. Many people with TMD also live with other chronic overlapping pain conditions (COPCs) such as fibromyalgia, irritable bowel syndrome, or chronic fatigue syndrome.

Depression, anxiety, sleep problems, and persistent cognitive impairments are concomitant health issues associated with painful TMDs [8, 9]. Combined with the pain and functional limitations that accompany TMDs, these health issues can significantly lower an individual’s quality of life and interfere with activities of daily living [10, 11]. Large studies, including the Orofacial Pain Prospective Evaluation and Risk Assessment (OPPERA) study, have shown that comorbid physical symptoms, central sensitization, psychosocial factors, and altered autonomic nervous system function all play important roles in how TMDs develop and affect people [12, 13].

Assessment and treatment should extend beyond patient self-reports, which typically focus on pain and related physical symptoms. Comprehensive assessment that also considers psychophysiological indicators, such as VOCL biomarkers, could provide valuable insight into how pain is experienced and expressed.

TMD represents a heterogeneous group of disorders rather than a single diagnostic entity [3, 14, 15]. Clinical presentations of TMDs include myogenous TMD, characterized primarily by masticatory muscle pain, and arthrogenous TMD, involving intra-articular joint pathology, disc displacement disorders, and degenerative joint disease. Additionally, patients vary in the duration of symptoms (acute versus chronic), the degree of central sensitization, and psychosocial burden [8, 16, 17, 18]. These distinctions are likely to influence VOCL domains differentially. For example, individuals whose symptoms are driven primarily by muscular involvement may experience reduced articulatory precision or slower diadochokinetic performance because of pain-related guarding. In contrast, individuals with more centrally mediated pain or greater psychosocial burden may show broader changes in prosody, speech timing, or cognitive-linguistic performance.

These differences are important to consider when exploring behavioral biomarkers, as variations in underlying pain mechanisms may lead to different patterns across the vocal, oropharyngeal, and cognitive-linguistic domains [19, 20]. Recognizing the heterogeneity of TMD will therefore be essential to developing and validating VOCL-based approaches to pain phenotyping.

## 3. Limitations of current TMD assessment

Current clinical assessment of temporomandibular disorders (TMDs) relies predominantly on patient-reported pain intensity and symptom severity measures, most commonly the Visual Analog Scale (VAS) and the Numeric Pain Rating Scale (NPRS) [21, 22].

However, when used in isolation, these commonly applied pain assessment tools provide a limited representation of an individual’s pain experience. Important dimensions such as the emotional, qualitative, and functional aspects of pain are often not adequately assessed [23]. Moreover, while self-report measures are available to assess the potential impact of pain on essential activities of daily living, such as eating, speaking, or sleeping, the direct characterization of VOCL function has not been incorporated into TMD assessment [15, 24]. It has been recommended that chronic pain assessments transition toward multidimensional approaches that integrate patient-reported outcomes with psychological and functional measures, thereby providing a more comprehensive characterization of pain [25, 26, 27]. Consistent with these recommendations, we propose that VOCL biomarkers represent a potentially important enhancement to the contemporary TMD assessment framework.

## 4. Biomarkers in TMD—current gaps

Existing research on TMD-related biomarkers has primarily focused on biological measures such as neuroimaging and inflammatory mediators (*e.g.*, cytokines and neuropeptides) measured in blood, saliva, or other tissues. These biomarkers have the capability to reveal mechanistic information about pain-related inflammatory and brain processes, but they are invasive, costly, and often impractical in clinical settings [28, 29]. They also fail to capture dynamic behavioral expressions of pain, such as vocal strain, reduced prosody, cognitive deficits, or swallowing impairment, which are dimensions that VOCL biomarkers could address.

## 5. The case for vocal, oropharyngeal, and cognitive-linguistic (VOCL) biomarkers

Chronic TMD pain affects multiple body systems, including the oral and pharyngeal anatomical mechanisms involved in controlling voice, speech, swallowing, and language [15, 30].

Beyond physical discomfort, pain modulates neuromuscular control and autonomic activity that underlie phonation and articulation. Even mild or persistent pain can alter laryngeal tension, respiratory drive, and supraglottic coordination, producing subtle but measurable changes in tone, pitch, prosody, and speech rate [31]. A vocal biomarker is a set of features extracted from an audio signal that are linked to a clinical outcome, used for monitoring, diagnosis, or severity grading [32, 33, 34]. Recent studies support the sensitivity of vocal parameters to pain-related physiological and affective states. Evidence has shown that experimentally induced pain is associated with decreased fundamental frequency variability and altered spectral energy distribution, reflecting changes in muscle tension and breath support. Pain may also influence vocal intensity and phonation duration through sympathetic arousal, which modifies subglottal pressure and vocal fold stiffness [35, 36, 37]. These findings align with neurophysiological models suggesting that pain and vocalization share overlapping pathways within the limbic and brainstem systems, integrating emotional and autonomic control of voice production. This convergence provides a strong rationale for exploring voice as a dynamic, non-invasive biomarker of pain [38]. Vocal metrics, when paired with cognitive-linguistic and oropharyngeal assessments, could offer a multidimensional window into how chronic pain impacts communication and behavioral function, complementing self-reported measures and potentially improving phenotyping and treatment monitoring in pain populations [39, 40].

Vocal biomarkers may be particularly relevant for characterizing pain in individuals with TMD for several reasons (see Fig. 1). A summary of the VOCL domains, example assessment tasks, and candidate metrics is provided in Table 1.

**Fig. 1.** Conceptual framework examining potential associations between TMD-related manifestations and VOCL indicators. This figure illustrates areas that might be impacted in TMDs, their associated functional, cognitive, and behavioral manifestations, and the applicability of VOCL biomarkers in monitoring these changes. The black pathway depicts some of the ways a TMD can affect patients. The blue pathway outlines how TMD manifests within each domain. The red pathway identifies the features that can be extracted from vocal recordings. The green pathway highlights the physiological processes and cognitive-behavioral outcomes that can be evaluated through these vocal features. ROM: Range of Motion; MOC: Memory, Organizational, and Comprehension; TMD: Temporomandibular disorder; DDK: Diadochokinetic.

**Table 1.** Overview of vocal, oropharyngeal, and cognitive-linguistic (VOCL) domains, example assessment tasks, and candidate acoustic or behavioral metrics proposed for investigation in temporomandibular disorder (TMD) research.

VOCL Domain	Example Assessment Tasks	Candidate Acoustic/Behavioral Metrics
Vocal/Voice Acoustics (Laryngeal & Respiratory Control)	• Sustained vowel phonation (*e.g.*, /a/) • Standardized oral reading passages • Voice Handicap Index (VHI) questionnaire	• Fundamental frequency (F_0_) and F_0_ variability • Jitter (cycle-to-cycle frequency variation) • Shimmer (amplitude variation) • Harmonics-to-noise ratio (HNR) • Spectral slope and subharmonics components • Speech–pause ratio • Mean phonation time
Motor Speech	• Diadochokinetic (DDK) tasks (*e.g.*, /pa/, /ta/, /ka/, /pataka/) • Connected speech samples • Syllable repetition tasks	• DDK rate (syllables/second) • Articulatory precision and range of motion • Speech rate (words/minute) • Prosodic measures (rhythm, stress, intonation)
Oropharyngeal Function	• Standardized swallowing tasks (liquid and solid bolus) • Clinical mastication assessment • Maximal interincisal opening (MIO) measurement	• Oral transit time (OTT) • Piecemeal deglutition frequency • Maximal interincisal opening (mm) • Self-rated swallowing effort (Borg/VAS scale)
Cognitive-Linguistic	• Digit span and word span tasks • Verbal fluency tasks (category-based & letter-based) • Multi-step direction-following tasks • Story retelling and narrative comprehension tasks	• Forward and backward span scores • Fluency: total words generated per category or letter • Narrative accuracy and omission rate • Response latency and error rate
Spontaneous and Conversational Speech (Naturalistic Behavior)	• Semi-structured or free conversation sampling • Narrative elicitation tasks • Ecological momentary assessment via smartphone	• Words per minute; conversational turn length • Pause frequency and mean pause duration • Disfluency rate (fillers “um”/“uh”, repetitions) • Lexical diversity (type–token ratio) • Negative affect or pain-related word frequency

*VHI: Voice Handicap Index; F_0_: fundamental frequency; HNR: harmonics-to-noise ratio; DDK: diadochokinetic; VAS: Visual Analog Scale. All metrics are proposed candidates for future validation research in TMD populations.*

## 6. Lessons from other conditions

Biomarkers derived from swallowing, oropharyngeal function, cognition, and voice have demonstrated value in other neurological and psychiatric disorders and even offered insight into both motor and cognitive status. An overview of current evidence from other conditions is summarized in Table 2. For example, increased jitter and shimmer in acoustic voice measures can reliably distinguish individuals with Parkinson’s disease from healthy controls [41, 42]. In major depressive disorder, reduced pitch variability has been linked to greater symptom severity [43, 44, 45]. In amyotrophic lateral sclerosis (ALS), slowed diadochokinetic (DDK) rates and changes in swallowing biomechanics have been used to detect early bulbar involvement [46]. Cognitive-linguistic measures, such as reduced verbal fluency, have also been associated with disease progression in multiple sclerosis and Parkinson’s disease [42, 47, 48]. These findings illustrate how subtle changes across multiple domains such as speech acoustics, swallowing physiology, and cognitive-linguistic performance can serve as sensitive, non-invasive indicators of disease status. Applying similar multidimensional biomarker strategies to TMD could provide a low-burden way to phenotype patients, monitor changes over time, and guide precision pain management.

**Table 2.** Overview of current evidence regarding utilization of vocal, oropharyngeal, and cognitive-linguistic (VOCL) biomarkers in other conditions.

VOCL Domain	Evidence in TMD (Illustrative)	Evidence in Other Conditions (Illustrative)	Current Validation Level*
Vocal Acoustics/Voice	Limited observational studies report altered voice quality and voice-related QoL in individuals with TMD, with worse scores associated with greater TMD severity and jaw functional limitation.	Extensive work in Parkinson’s disease, depression, and other conditions shows that F_0_, jitter, shimmer, HNR, and related features are sensitive to disease status and affective states.	Exploratory in TMD; preliminary to moderate in other conditions.
Motor Speech	Small studies demonstrate altered mandibular movement patterns during speech and potential articulatory adaptations in TMD.	In ALS and other neurologic disorders, DDK rate, articulatory precision, and prosodic changes are established markers of bulbar involvement and dysarthria.	Exploratory in TMD; early clinical utility in neurology.
Oropharyngeal/Swallowing	Several studies document chewing and swallowing dysfunction, prolonged oral transit time, and tongue/masticatory weakness in chronic TMD.	Dysphagia assessment is well-validated in stroke, neurodegenerative disease, and geriatric populations, with robust clinical protocols and outcome measures.	Emerging in TMD; clinically established in dysphagia care.
Cognitive–Linguistic Function	Initial work suggests that patients with painful TMD report cognitive complaints and may show subtle objective deficits on selected tasks.	Systematic reviews in chronic pain, depression, and Parkinson’s disease show consistent associations between pain/mood and working memory, attention, and verbal fluency performance.	Very limited in TMD; moderate evidence in other pain and neurologic conditions.
Spontaneous/Conversational Speech	No direct TMD-specific datasets yet, but conceptual rationale and early speech–pain research suggest feasibility of naturalistic speech markers.	Studies in neuropharmacology and other clinical populations show that disfluency, rate, and lexical patterns can index cognitive or medication-related changes.	Conceptual only in TMD; early-stage research elsewhere.

*Validation levels: Exploratory: proof-of-concept only; Emerging: initial empirical support; Established: validated clinical protocols available. F_0_: fundamental frequency; HNR: harmonics-to-noise ratio; DDK: diadochokinetic; ALS: amyotrophic lateral sclerosis; QoL: quality of life; VOCL: vocal, oropharyngeal, and cognitive-linguistic; TMD: temporomandibular disorder.

## 7. Vocal/voice acoustics (laryngeal & respiratory control)

Pain and muscular guarding are frequently seen in individuals with TMD and extend beyond the jaw and facial muscles. These muscle changes can influence laryngeal and respiratory control, resulting in altered vocal stability and resonance [49]. Although limited research has examined voice changes in individuals with TMD, individuals with TMD have shown reduced voice quality on cepstral analysis compared to healthy controls, and greater TMD severity was correlated with lower cepstral measurement values (*rho* = −0.57 to −0.88, *p* < 0.001) [50], indicating poorer voice quality in those with more severe TMD. Acoustic features such as fundamental frequency (F_0_), which specifically represents vocal pitch, may become less stable under conditions of pain and stress. Perturbation measures, such as jitter (cycle-to-cycle frequency variation) and shimmer (loudness variation), are elevated in conditions involving increased muscular tension, such as muscle tension dysphonia, and are sensitive indicators of disrupted vocal fold vibration induced by excess muscle activity [51, 52]. Meanwhile, the harmonics-to-noise ratio (HNR) has been linked to voice quality and provides information regarding the degree of breathiness or strain in phonation. Additional metrics like spectral slope and subharmonics further reflect irregular or strained vocal fold vibrations, while temporal features such as speech–pause ratio and mean phonation time provide insight into how pain may reduce respiratory support for sustained speech [53]. Collectively, these acoustic measures offer noninvasive and objective means to quantify how TMD-related pain and tension impact vocal control and stability.

## 8. Motor speech in TMD

Speech is a complex neuromuscular fine motor process wherein the brain smoothly coordinates respiration, phonation, resonance, articulation, and prosody. Muscular dysfunction interrupting any of these subsystems can lead to impairments in intelligibility, naturalness, and effective communication [54]. Motor speech assessment is an important but commonly overlooked component of TMD evaluation. Jaw pain, restricted mandibular movement, and compensatory muscular patterns are often associated with TMD and can directly influence precise articulation and vocal quality. Clinically, speech-language pathologists conduct motor speech evaluations using connected speech samples, sustained phonation, and diadochokinetic (DDK) rates to identify any restrictions in articulatory and linguistic range of motion, coordination, and timing of jaw and tongue movement [55]. Motor speech biomarkers, such as DDK rates, could be particularly informative for evaluating individuals with TMD because they directly assess the speed, precision, and coordination of the articulatory system. These speech assessment tasks require rapid, alternating movements of the lips, tongue, and jaw structures, which may be meaningfully affected in TMD due to pain, muscle tension, or restricted range of motion. In neurological conditions like ALS, slowed DDK rates signal bulbar dysfunction, and similar performance decrements in TMD could reveal pain-related alterations in motor control [46]. Including DDK tasks in TMD assessment allows us to capture these subtle motor impairments that may not be evident in conversational speech but are highly relevant to functional outcomes. Research has demonstrated that patients with TMD exhibit altered mandibular movements during speech compared to controls, demonstrating compensatory or restricted motor patterns that may affect clarity and efficiency of articulation [56].

## 9. Oropharyngeal function in TMD

Mastication (chewing) and deglutition (swallowing) require coordination of temporomandibular joint motion and orofacial muscle function. In individuals with TMD, pain and restricted jaw movement can disrupt normal chewing patterns, increasing the risk of inefficient bolus preparation and subsequent swallowing difficulties, including dysphagia and aspiration-related complications [57]. As a consequence, oral transit time (OTT), defined as the time it takes for food to move from the oral cavity to the pharynx, can become prolonged. Piecemeal deglutition, the need for repeated swallows to adequately clear the oropharynx after a single swallow, can suggest effortful swallowing possibly due to inadequate mastication of the bolus of food [58, 59]. Interestingly, individuals with TMD frequently report elevated effort during chewing and swallowing tasks, and objective measures such as maximal interincisal opening provide evidence of this reduced jaw mobility [60]. Thus, oropharyngeal markers may be highly relevant to TMD because they reflect the everyday functional demands placed on the jaw during eating. Implementing swallow-based biomarkers into the assessment of TMD may therefore provide valuable indicators of the extent to which TMD pain disrupts one of the most fundamental oral motor activities.

## 10. Cognitive-linguistic function in TMD

The effects of TMD are not limited to musculoskeletal function but may extend to cognition and language through pain–cognition interactions [61]. Staneszewski and colleagues reported that, although individuals with TMD may demonstrate preserved neurocognitive functions, they may report self-perceived cognitive difficulties that could interfere with coping with chronic pain and managing common everyday tasks [61]. Chronic pain competes for attentional resources, taxing working memory and reducing efficiency in language-related tasks [62]. For example, patients may show reduced capacity in digit or word span tasks, reflecting limited working memory. Following multi-step directions becomes more difficult as pain interferes with divided attention. Verbal fluency tasks, in which individuals generate words either by category or by initial letter, may show declines when pain or sleep disruption diminishes cognitive flexibility [63, 64, 65, 66]. Tasks that involve story retelling demand integration of comprehension, memory, and organization. Evidence from chronic pain populations indicates that heightened pain can increase omissions and reduce accuracy in such tasks, raising the possibility that TMD-related pain may similarly interfere with narrative performance [65]. These cognitive-linguistic measures demonstrate how chronic TMD pain can extend beyond the jaw to affect higher-order communication and cognitive processes, reflecting the broader neurocognitive burden of persistent pain.

## 11. Spontaneous and conversational speech (naturalistic behavior) in TMD

Finally, spontaneous and conversational speech provides a window into how TMD affects real-world communication. Unlike structured tasks, conversational speech reveals natural adaptations to pain in everyday interactions [27]. Patients in pain may produce fewer words per minute and shorter conversational turns, reflecting fatigue or discomfort [67]. Pauses may occur more frequently or last longer, while disfluencies such as fillers (“um”, “uh”) or repetitions can increase as pain interferes with fluency and flow [68]. Word choice may also shift, with greater use of negative affect or pain-related terms, while lexical diversity (the range of unique words used) may decrease, resulting in simpler and less varied language [27]. These markers are especially important because they demonstrate how chronic pain in TMD can alter everyday communication beyond controlled laboratory measures, providing ecologically valid biomarkers of pain burden and functional impact.

### 11.1 Potential confounders and standardization considerations

Voice, speech, swallowing, and cognitive-linguistic measures are sensitive to a range of biological, psychological, and environmental factors that must be considered when evaluating VOCL features in TMD [55]. Key biological confounders include age, sex, hormonal status, smoking history, respiratory conditions, neurological disorders, and medication use, particularly antidepressants and muscle relaxants. Psychological variables such as anxiety, depression, catastrophizing, and sleep disturbance are also known to influence vocal and cognitive performance and are highly prevalent in TMD populations [17, 69, 70].

To ensure reproducibility, minimum recording standards should be established. These include use of calibrated microphones, consistent microphone-to-mouth distance, controlled ambient noise levels, standardized speech and motor tasks, and documentation of pain state at the time of recording. Without rigorous control of these factors, observed acoustic or behavioral differences may reflect extraneous variability rather than TMD-related processes. Future investigations should incorporate systematic measurement and statistical adjustment of these variables to evaluate the specificity of VOCL features to TMD versus general chronic pain or affective states.

### 11.2 Measurement reliability and within-subject variability

Before clinical translation, VOCL measures must demonstrate acceptable reliability and stability. Chronic pain conditions are characterized by symptom fluctuation, and within-subject variability during pain fluctuations must be quantified [71]. Studies should report test–retest reliability metrics, such as intraclass correlation coefficients (ICCs), and estimate minimal detectable change thresholds to determine whether observed differences exceed measurement error and represent meaningful changes [72, 73]. Longitudinal designs are particularly important for determining whether VOCL features track changes in pain intensity or functional status over time. Without demonstrating reliability and responsiveness, interpretation of VOCL alterations remains challenging.

## 12. Discussion

The literature synthesized in this review suggests that chronic TMD may influence vocal, oropharyngeal, and cognitive-linguistic domains through neuromuscular, autonomic, and cognitive mechanisms. However, direct empirical evidence within TMD populations remains limited. At present, VOCL measures should be regarded as investigational rather than clinically established biomarkers.

Current TMD assessment relies predominantly on patient-reported outcomes, which capture pain intensity and perceived burden but provide limited insight into functional communication and behavioral expression. VOCL measures may represent a potential approach for investigating these dimensions, particularly when integrated with psychological and physiological data. Nevertheless, rigorous validation is required before conclusions regarding diagnostic accuracy, treatment monitoring, or clinical utility can be drawn.

## 13. Proposed validation pathway for VOCL biomarkers in temporomandibular disorders

A staged validation process will be necessary before VOCL features can be considered reliable behavioral biomarkers in temporomandibular disorders. Establishing VOCL biomarkers in temporomandibular disorders will require systematic validation across multiple stages. Initial work should characterize VOCL features in individuals with TMD compared with healthy populations while accounting for known biological and psychosocial confounders. Subsequent research may examine whether patterns differ across clinically relevant TMD presentations, including muscular and intra-articular conditions, as well as varying levels of chronicity and psychosocial burden. Longitudinal studies will also be important for determining whether VOCL features correspond to fluctuations in pain severity, functional limitations, or treatment response. Together, these approaches may help clarify the potential role of VOCL measures in future research on TMD pain phenotyping.

## 14. Clinical and research significance

VOCL features represent a promising but currently underexplored dimension of temporomandibular disorder research. Unlike traditional biomarkers such as neuroimaging or inflammatory markers, VOCL measures can be readily incorporated into clinical and telehealth environments. Evidence from neurological and psychiatric conditions demonstrates that subtle changes in voice, speech motor control, swallowing function, and cognitive-linguistic performance can reflect underlying disease processes. Applying these concepts to TMD may provide new opportunities to study functional manifestations of pain and to complement existing patient-reported outcomes with objective behavioral measures.

## 15. Limitations and future research directions

Most of the current evidence for VOCL biomarkers derives from studies of neurological and psychiatric disorders rather than individuals with TMD. Hence, there is a clear need for research applying VOCL biomarkers in people with TMD [34]. Specifically, research involving diverse TMD populations is needed to validate their reliability and refine their integration into future research and clinical practice [74, 75]. In addition, multicenter collaborations would help to generate the large datasets required for machine learning model development, allowing for more equitable outcomes and more accurate results. Integration of VOCL biomarkers within existing psychological and physiological measures would help create comprehensive multimodal diagnostic systems. Mobile health and telehealth applications represent promising avenues through which these biomarkers could be more broadly implemented. Pursuing these research opportunities will help determine the value of VOCL biomarkers for assessing pain phenotypes in individuals living with TMD.

## 16. Conclusions

Temporomandibular disorders are heterogeneous, multisystem pain conditions that extend beyond localized musculoskeletal dysfunction [1]. Vocal, oropharyngeal, and cognitive-linguistic features represent promising candidate domains for investigating behavioral correlates of TMD-related pain. However, empirical evidence specific to TMD remains limited, and these measures should currently be considered investigational. Establishing standardized protocols, addressing potential confounders, and implementing staged validation studies will be essential before clinical integration can be considered. With rigorous methodological development, VOCL measures may help clarify functional manifestations of TMD and contribute to emerging precision pain research frameworks.
